# Reduced Temporal Muscle Thickness Is Associated with Increased Postoperative Complications After Cranioplasty

**DOI:** 10.3390/jcm15134997

**Published:** 2026-06-26

**Authors:** Arina V. Blehm, Artem Rafaelian, Silvia Hernandez-Duran, Thomas M. Freiman, Peter Baumgarten, Thomas Freitag, Florian Gessler, Daniel Dubinski, Sae-Yeon Won

**Affiliations:** 1 Department of Neurosurgery, University Medicine Rostock, 18057 Rostock, Germany; arinablehm@googlemail.com (A.V.B.);; 2 Department of Neurosurgery, University Hospital Augsburg, 86156 Augsburg, Germany

**Keywords:** cranioplasty, temporalis muscle thickness, postoperative complications, frailty, decompressive craniectomy

## Abstract

**Background/Objectives**: Cranioplasty is a common reconstructive procedure following decompressive craniectomy, yet postoperative complications requiring reoperation remain frequent. Sarcopenia has been associated with adverse surgical outcomes. Temporalis muscle thickness (TMT), readily assessed on routine cranial CT, has been proposed as a surrogate marker of sarcopenia; however, its role in predicting cranioplasty outcomes remains to be established. This study aimed to evaluate the association between TMT and postoperative complications requiring reoperation after cranioplasty. **Methods**: In this retrospective single-center cohort study, 71 patients undergoing cranioplasty after decompressive craniectomy were included. Patients were stratified according to the occurrence of postoperative complications requiring reoperation into a complication group (*n* = 28) and an uneventful postoperative course group (*n* = 43). TMT was measured on preoperative CT scans obtained prior to craniectomy and prior to cranioplasty. Reduced TMT was defined as ≤5 mm. **Results**: Postoperative complications requiring surgical revision occurred in 39.4% of patients. Reduced TMT (≤5 mm) was significantly associated with greater reoperation risk in univariate analysis (*p* = 0.003). Patients undergoing surgical revision had significantly lower TMT prior to craniectomy (4.6 mm vs. 5.3 mm; *p* = 0.03) and TMT remained an independent predictor in multivariate analysis. **Conclusions**: Reduced TMT is independently associated with an increased risk of postoperative complications after cranioplasty requiring surgical revision and may serve as a simple imaging-based marker for preoperative risk stratification.

## 1. Introduction

Temporalis muscle thickness (TMT) has emerged as a readily accessible imaging-based marker for predicting outcomes after neurosurgical procedures and is increasingly regarded as a surrogate marker of sarcopenia due to its ease of assessment on routine cranial CT imaging [1]. Reduced TMT, often reflecting underlying sarcopenia, has been associated with poorer recovery and unfavorable outcomes in patients undergoing surgery for stroke, traumatic brain injury (TBI), or head and neck cancer [2,3,4].

Decompressive craniectomy (DC) is commonly performed in patients with TBI or acute ischemic stroke (AIS) to relieve elevated intracranial pressure caused by cerebral edema, as well as in cases of acute subdural or epidural hematomas requiring surgical decompression [5,6,7]. After stabilization, cranioplasty is performed either by reimplantation of the autologous bone flap or by insertion of a patient-specific implant [8]. The optimal timing of cranioplasty has not been established and depends largely on the individual clinical condition of the patient, with reported intervals ranging from 30 to 90 days [9]. Postoperative complications occur in up to 40% of cases. The most frequent complications include infection, bone flap resorption and hemorrhage, often necessitating revision surgery [9,10]. In addition, neurological symptoms such as seizures, headaches, or behavioral changes have been described in patients with craniectomy defects, with the so-called “sinking flap syndrome” representing a possible pathophysiological mechanism [10,11,12].

Several factors have been identified as predictors of outcome after DC and cranioplasty, including low Glasgow Coma Scale (GCS) score, significant midline shift, advanced age, and pre-existing comorbidities such as diabetes mellitus or cardiovascular disease. However, reliable and easily obtainable radiological markers reflecting a patient’s physiological reserve are still lacking [13,14,15]. TMT can be readily measured on standard cranial CT scans without additional imaging or cost. As a surrogate marker of sarcopenia, it may reflect the patient’s overall physiological reserve. Despite increasing evidence linking reduced TMT to poorer outcomes in various neurosurgical populations, its role in predicting postoperative complications after cranioplasty has not been sufficiently investigated [1,2,3,4]. Therefore, this study aimed to evaluate whether reduced preoperative TMT is associated with an increased risk of postoperative complications requiring reoperation after cranioplasty.

## 2. Methods

**Figure 1:**
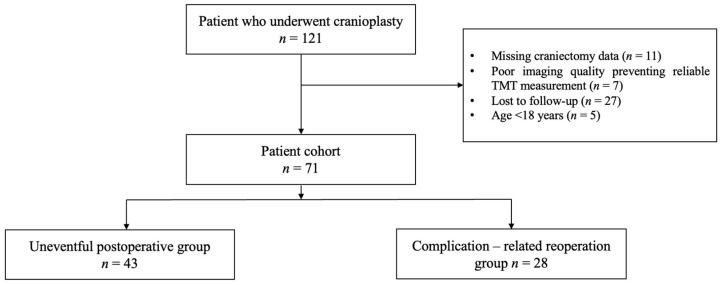
Flowchart of patient selection and study cohort allocation. A total of 121 patients who underwent cranioplasty after decompressive craniectomy were screened for eligibility. Fifty patients were excluded because of missing craniectomy data (*n* = 11), poor imaging quality preventing reliable TMT assessment (*n* = 7), loss to follow-up (*n* = 27), or age < 18 years (*n* = 5).

This retrospective single-center cohort study was conducted at the Department of Neurosurgery, University Medicine Rostock, Germany. Between January 2018 and December 2025, a total of 121 patients who underwent decompressive craniectomy followed by subsequent cranioplasty were screened for eligibility. Seventy-one patients met the predefined inclusion criteria and were included in the final analysis (Figure 1). The study aimed to evaluate whether TMT, as a potential surrogate marker of sarcopenia, could be associated with postoperative complications following cranioplasty.

Ethical approval was obtained from the Ethics Committee of the University Medicine Rostock, Germany (Identification number: A 2020-0296). Due to the retrospective and non-interventional study design, the requirement for informed consent was waived. Patients who underwent cranioplasty following decompressive craniectomy between January 2018 and December 2025 were screened for eligibility. Inclusion criteria were: (1) availability of preoperative cranial CT imaging obtained prior to decompressive craniectomy, (2) availability of preoperative cranial CT imaging obtained prior to cranioplasty, and (3) complete clinical documentation including postoperative follow-up data. Patients with bilateral decompressive craniectomy, unavailable imaging suitable for TMT assessment, or missing follow-up data were excluded from the analysis.

Clinical data were retrospectively collected from electronic medical records. The following variables were analyzed: Age and sex; Underlying diagnosis leading to decompressive craniectomy; Comorbidities; Medication history, including anticoagulant and antiplatelet therapy; Time interval between craniectomy and cranioplasty; and TMT prior to craniectomy and prior to cranioplasty.

Patients were divided into two groups: uneventful postoperative course group and complication-related reoperation group. The primary endpoint was defined as postoperative complications requiring surgical revision after cranioplasty. No minimum follow-up duration was required for inclusion. The duration was calculated from the date of admission for craniectomy to the last documented clinical or radiological data in the institutional medical record system.

Complications were not restricted to a predefined time interval and were categorized for descriptive purposes into: Early (<30 days) and late (>30 days).

### 2.1. Measurement of Temporalis Muscle Thickness

TMT was measured using the institutional PACS software (JiveX^®^ v5.2, VISUS Technology Transfer GmbH, Bochum, Germany). All measurements were performed independently by two neurosurgeons (A.V.B. and A.R.), each blinded to clinical data, to ensure unbiased assessment. Interobserver reliability was quantified using the intraclass correlation coefficient (ICC). Agreement between raters was excellent (ICC 0.96, 95% CI 0.94–0.98), with a mean absolute difference of 0.04 mm between observers.

**Figure 2:**
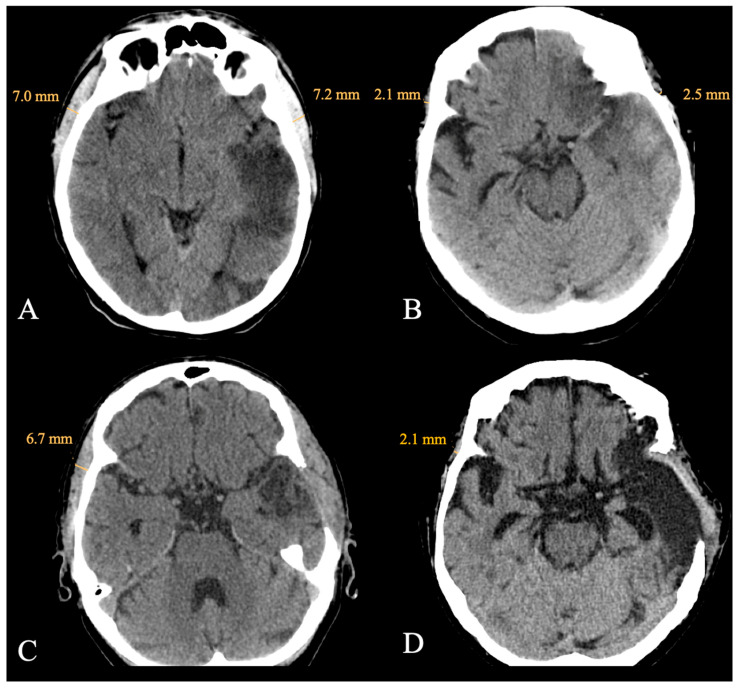
Representative axial cranial CT images demonstrating assessment of temporalis muscle thickness (TMT). (**A**) Patient with preserved TMT prior to decompressive craniectomy (mean bilateral TMT = 7.1 mm). (**B**) Patient with reduced TMT prior to decompressive craniectomy (mean bilateral TMT = 2.3 mm). (**C**) Same patient as in (**A**) after decompressive craniectomy, showing preserved contralateral TMT prior to cranioplasty (contralateral TMT = 6.7 mm). (**D**) Same patient as in (**B**) after decompressive craniectomy, demonstrating persistently reduced contralateral TMT prior to cranioplasty (contralateral TMT = 2.1 mm).

Measurements were obtained on cranial CT slices reconstructed using a standard soft-tissue kernel and standardized soft-tissue window settings. TMT was assessed on preoperative CT scans obtained prior to decompressive craniectomy as well as on imaging performed before cranioplasty. Pre-craniectomy TMT measurements were obtained from cranial CT scans acquired immediately before decompressive craniectomy, prior to decompressive craniectomy, TMT was measured bilaterally and the mean value was used. Prior to cranioplasty, measurements were obtained from the contralateral side only to avoid distortion by surgical manipulation. The temporalis muscle was visualized in a horizontal orientation approximately 5 mm above the superior orbital wall. TMT was measured on the contralateral (unaffected) side. For imaging obtained prior to cranioplasty, measurements were also performed on the contralateral (unaffected) side to avoid potential bias related to surgical manipulation or muscle atrophy on the operated side (Figure 2). The median cohort TMT was used as an exploratory dichotomization threshold.

### 2.2. Statistical Analysis

Statistical analysis was performed using SPSS Statistics (IBM Corp., Version 29.0.1.1, Armonk, NY, USA) and GraphPad Prism (GraphPad Software, Version 10.5.0, San Diego, CA, USA). Descriptive statistics were calculated using Microsoft Excel (Microsoft Corporation, Version 16.109, Redmond, WA, USA). Continuous variables are presented as median and interquartile range (IQR), and categorical variables as absolute numbers and percentages. Between-group comparisons were performed using the Mann–Whitney U test for continuous variables and appropriate tests for categorical variables. Univariate logistic regression analysis was conducted to identify factors associated with postoperative complications requiring reoperation. Given the limited number of outcome events, a parsimonious multivariable logistic regression model including age, sex, baseline modified Rankin Scale (mRS), continued acetylsalicylic acid therapy, and TMT prior to decompressive craniectomy was constructed. TMT was analyzed as a continuous variable. Odds ratios (ORs) with 95% confidence intervals (CIs) were calculated. As no universally accepted TMT cutoff exists, an exploratory analysis using the cohort median value of 5 mm was additionally performed for descriptive comparisons. Postoperative complications were categorized as early (<30 days) or late (>30 days). A two-sided *p*-value < 0.05 was considered statistically significant.

## 3. Results

A total of 71 patients were included in the final analysis. The median age was 61 years (IQR 51–67), and 26 patients (36.6%) were female. The most common indication for decompressive craniectomy was stroke (45.1%), followed by traumatic brain injury (23.9%), hemorrhage (19.7%), and infection (9.9%) (Table 1). Median BMI was 25.5 kg/m^2^ (IQR 22.4–29.7). The median ASA score was 3 (IQR 3–3). At baseline, most patients presented with moderate to severe disability (mRS 3–4 in 50.7%). The median interval between craniectomy and cranioplasty was 116 days (IQR 97–184).

Median TMT prior to craniectomy was 5.0 mm (IQR 4.3–5.6). As this corresponded to the median value of the cohort, 5 mm was used as a descriptive cutoff to categorize patients into two groups (≤5 mm vs. >5 mm) for further analysis. Prior to craniectomy, 38 patients (53.5%) had TMT > 5 mm, whereas 33 patients (46.5%) had TMT ≤ 5 mm.

Prior to cranioplasty, median TMT was 4.3 mm (IQR 3.8–5.0), with 66.2% of patients demonstrating TMT ≤ 5 mm. A reduction in muscle thickness between craniectomy and cranioplasty was observed in 80.2% of cases.

Nineteen patients (26.7%) underwent CSF shunt surgery, and 4 patients (5.6%) presented with pre-existing sinking flap syndrome. Autologous bone grafts were used in the majority of cases (83.1%). Continued acetylsalicylic acid therapy prior to cranioplasty was documented in 25.4% of patients. At discharge, functional outcome remained predominantly moderate (mRS 3–4 in 50.7%), while 2 patients (2.8%) had died (mRS 6).

The median follow-up duration was 369 days (IQR 147.7–702.2). Follow-up duration was comparable between patients with TMT > 5 mm (328.5 days, IQR 138–849.5) and those with TMT ≤ 5 mm (361 days, IQR 150–644).

For outcome analysis, the cohort was divided into two groups: patients who developed postoperative complications requiring surgical revision after cranioplasty and those with an uneventful postoperative course not requiring reoperation. Overall, 28 patients (39.4%) underwent complication-related revision surgery, whereas 43 patients (60.6%) experienced no postoperative complications requiring further surgical intervention. There were no significant differences between groups regarding age, sex, BMI, ASA score, baseline mRS, time interval between surgeries, operative duration, decompressive craniectomy area, implant material, or major comorbidities (Table 2).

Median TMT prior to decompressive craniectomy was significantly lower in patients requiring reoperation compared with those with an uneventful postoperative course (4.6 mm vs. 5.3 mm, *p* = 0.03). As an exploratory analysis, patients were additionally categorized according to the cohort median TMT of 5 mm. Patients with a TMT ≤ 5 mm prior to decompressive craniectomy showed a significantly higher rate of postoperative complications than those with a TMT > 5 mm (57.6% vs. 23.7%; OR 4.37, 95% CI 1.58–12.10; *p* = 0.003). This corresponded to an absolute risk difference of 33.9 percentage points.

Although median pre-cranioplasty TMT did not differ significantly between groups, exploratory categorization using the 5 mm cutoff was associated with postoperative complications in univariate analysis (OR 3.64, 95% CI 1.16–11.37; *p* = 0.019).

In the parsimonious multivariable logistic regression model, which included age, sex, baseline modified Rankin Scale (mRS), continued acetylsalicylic acid therapy, and pre-craniectomy TMT, lower TMT remained independently associated with complication-related reoperation. For each 1 mm decrease in pre-craniectomy TMT, the odds of reoperation increased by a factor of 2.08 (95% CI 1.16–3.70; *p* = 0.014) (Table 3).

Among the 28 patients requiring surgical revision, early complications (<30 days) occurred in 11 patients (39.3%), whereas 17 patients (60.7%) developed late complications (>30 days). Bleeding or hematoma was exclusively observed in the early postoperative period and occurred in 7 patients (25%). Postoperative cerebral edema was detected in 2 patients (7.1%), both classified as early complications. Flap dislocation was rare and occurred in one patient (3.6%), also within 30 days after cranioplasty (Table 4).

In contrast, infectious complications were predominantly late events, occurring in 7 of 8 cases (41.2% of late complications). Aseptic bone flap necrosis was exclusively observed as a late complication and accounted for 8 cases (28.6%), representing one of the most frequent causes of delayed revision surgery. Wound healing disorders were recorded in 2 patients (7.1%), both occurring in the late postoperative period. Re-craniectomy was required in 14 patients (50%), representing the most common revision procedure. Revision surgery without implant removal was performed in 8 patients (28.6%), predominantly in the early postoperative period (63.6%).

## 4. Discussion

The principal finding of this study is that reduced TMT prior to decompressive craniectomy was independently associated with postoperative complications requiring surgical revision after cranioplasty. Patients with a TMT ≤ 5 mm showed a substantially higher risk of complication-related reoperation than patients with a TMT > 5 mm. These findings suggest that preoperative TMT may serve as a simple imaging-based marker of reduced physiological reserve in patients undergoing cranioplasty. One possible explanation for the stronger association of pre-craniectomy TMT with postoperative complications is that this measurement may better reflect the patient’s baseline physiological reserve before major neurosurgical intervention. In contrast, TMT assessed before cranioplasty may already be influenced by surgery-related changes, including muscle atrophy, scarring, altered anatomy, or denervation. Moreover, pre-craniectomy TMT was assessed bilaterally, whereas pre-cranioplasty TMT was measured on the contralateral side to avoid distortion from the operated side.

With regard to cranioplasty-specific risk factors, several studies have identified variables associated with postoperative complications. Jin et al. demonstrated in a retrospective cohort that previous temporalis muscle resection was significantly associated with surgical site infection and bone flap resorption, emphasizing the importance of muscle preservation [16]. Similarly, Kim et al. identified prolonged operative time, preoperative subgaleal fluid collections, postoperative wound disruption, and temporalis muscle resection as significant predictors of graft infection requiring removal [17].

Beyond cranioplasty, TMT has shown prognostic relevance across various neurological conditions. Higher TMT has been associated with favorable outcomes in acute ischemic stroke and functional recovery [18], whereas no consistent association has been observed in intracerebral hemorrhage [19]. In traumatic brain injury, however, TMT has again been linked to clinical outcomes [20]. These findings support the concept that TMT reflects overall physiological reserve, which may be diminished across different neurosurgical populations [21]. In line with this, TMT has been validated as a reliable marker of sarcopenia in patients with glioblastoma and may contribute to individualized therapeutic decision-making [22].

In the context of cranioplasty, patients are exposed to a second major surgical intervention, which is associated with systemic stress responses, inflammation, and impaired wound healing. Reduced TMT may therefore reflect diminished physiological reserves and increased susceptibility to postoperative complications [10,23,24,25,26,27]. Importantly, our findings suggest that TMT measured prior to decompressive craniectomy may better capture baseline physiological status than measurements obtained prior to cranioplasty. The latter may already be influenced by surgery-related factors such as muscle atrophy, denervation, or altered biomechanics, thereby reducing its predictive value.

Importantly, postoperative complications requiring reoperation represent a heterogeneous endpoint. In our cohort, early complications were predominantly hemorrhage and cerebral edema, whereas late complications mainly consisted of infection, wound healing disorders, and aseptic bone flap necrosis. Reduced TMT may be more closely related to impaired wound healing, nutritional reserve, and susceptibility to delayed complications than to early postoperative hemorrhage. Therefore, the observed association between TMT and complication-related reoperation likely reflects multiple underlying biological mechanisms. Owing to the limited number of events within each subgroup, separate analyses of individual complication categories were considered exploratory and should be interpreted with caution.

The lack of standardized thresholds for defining low TMT remains a major limitation in current research. Reported cut-off values vary considerably across studies, with sex-specific thresholds ranging from 2.78 to 5.2 mm in women and from 3.83 to 6.3 mm in men [28,29]. Although efforts have been made to establish standardized values, these remain influenced by patient-specific factors such as age and sex [30]. According to the European Working Group on Sarcopenia in Older People (EWGSOP), sarcopenia is defined using sex-specific cut-offs based on deviations from normative reference values, which may provide a conceptual framework for interpreting TMT. However, due to the heterogeneity of available thresholds and the relatively small sample size of our cohort, we applied a data-derived cut-off of 5 mm to facilitate clinical applicability while minimizing the risk of overfitting [30,31].

It should be emphasized that TMT cannot be considered a standalone diagnostic tool for sarcopenia. Comprehensive assessment requires evaluation of muscle strength, overall muscle mass, and physical performance [27]. Nevertheless, TMT offers a practical and readily accessible imaging-based marker. Its measurement, however, may be influenced by interobserver variability and methodological differences, underscoring the need for standardized protocols [28,32].

From a clinical perspective, early identification of patients with reduced physiological reserve may allow targeted interventions. Previous studies have demonstrated that nutritional optimization and structured physical activity can improve frailty and reduce adverse outcomes [33,34,35,36,37]. In this context, TMT may serve as a screening tool to identify high-risk patients who could benefit from such interventions. However, prospective studies integrating TMT assessment with structured rehabilitation strategies remain limited [38].

Future research should focus on prospective validation of TMT as a prognostic marker in cranioplasty patients, including its integration into multivariable risk models and its comparison with established clinical predictors. Standardization of measurement techniques and cut-off values will be essential to improve reproducibility and clinical applicability [39,40].

## 5. Limitations

This study is limited by its retrospective single-center design, relatively small sample size, and the exclusion of patients with missing imaging or follow-up data, which may have introduced selection bias. The sample size did not permit reliable evaluation of sex-specific TMT thresholds, although TMT is known to vary according to age and sex. TMT was assessed solely as an imaging-based surrogate marker of muscle status and may have been influenced by surgery-related changes. As no universally accepted TMT threshold exists, the 5 mm cutoff was derived from the cohort median and should be considered exploratory. Furthermore, the retrospective design did not allow for a predefined follow-up interval, potentially introducing time-at-risk bias for late complications. Finally, residual confounding by factors such as nutritional status, comorbidities, and perioperative management cannot be excluded.

## 6. Conclusions

Reduced TMT was associated with an increased risk of postoperative complications requiring reoperation after cranioplasty. These findings suggest that TMT may serve as an imaging-based marker of reduced physiological reserve and may support preoperative risk assessment. However, given the retrospective observational design, causality cannot be inferred. Prospective multicenter studies are required to validate these findings.

## Figures and Tables

**Table 1:** Demographics and outcome data.

Count	71
Age, median (IQR)	61 (51–67)
**Indication for craniectomy**	
Stroke, *n* (%)	32 (45.1)
Traumatic brain injury, *n* (%)	17 (23.9)
Infection, *n* (%)	7 (9.9)
Hemorrhage, *n* (%)	14 (19.7)
Others, *n* (%)	1 (1.4)
**Sex**	
Female, *n* (%)	26 (36.6)
BMI, kg/m^2^ median (IQR)	25.5 (22.4–29.7)
ASA, median (IQR)	3 (3–3)
**Baseline mRS**	
1–2, *n* (%)	23 (32.4)
3–4, *n* (%)	36 (50.7)
5, *n* (%)	12 (16.9)
Time between first and second surgery, median (IQR)	116 (97–184)
**TMT prior to craniectomy, median (IQR)**	5 (4.3–5.6)
>5 mm, *n* (%)	38 (53.5)
≤5 mm, *n* (%)	33 (46.5)
**TMT prior to cranioplasty, median (IQR)**	4.3 (3.8–5)
>5 mm, *n* (%)	24 (33.8)
≤5 mm, *n* (%)	47 (66.2)
Muscle thickness reduction between craniectomy and cranioplasty, *n* (%)	57 (80.2)
**CSF shunt surgery, *n* (%)**	19 (26.7)
Simultaneous, *n* (%)	3 (4.2)
Pre-existing sinking flap syndrome, *n* (%)	4 (5.6)
Operative duration (min), median (IQR)	107 (89–141)
Decompressive craniectomy area (cm^2^), median (IQR)	97.6 (81.7–109.2)
**Implant material**	
Autologous bone graft, *n* (%)	59 (83.1)
PEEK implant, *n* (%)	8 (11.3)
Titanium implant, *n* (%)	1 (1.4)
Synthetic implant (other alloplastic material), *n* (%)	3 (4.2)
Acetylsalicylic acid therapy, *n* (%)	29 (40.8)
Continued acetylsalicylic acid therapy prior to cranioplasty, *n* (%)	18 (25.4)
Antiepileptic drug therapy	54 (76.1)
Tracheostomy prior to cranioplasty	35 (49.3)
Alcohol abuse	10 (14.1)
Nicotine abuse	28 (39.4)
Type 2 diabetes mellitus	12 (16.9)
Obesity	14 (19.7)
Urinary tract infection, *n* (%)	8 (11.3)
**mRS at discharge**	
1–2, *n* (%)	20 (28.2)
3–4, *n* (%)	36 (50.7)
5, *n* (%)	13 (18.3)
6, *n* (%)	2 (2.8)
Follow-Up duration, median (IQR)	369 (147.7–702.2)
TMT > 5 mm, median (IQR)	328.5 (138–849.5)
TMT ≤ 5 mm, median (IQR)	361 (150–644)

Abbreviations: IQR: Interquartile range; TMT—Temporalis muscle thickness; BMI—Body Mass Index; mRS—modified Rankin Scale.

**Table 2:** Univariate analysis of juxtaposed characteristics with direct comparison of two patient-groups.

			Univariate		
	Uneventful Postoperative Course Group	Complication-Related Reoperation Group	OR	(CI 95%)	*p*-Value
**Count**	*n* = 43 (60.6)	*n* = 28 (39.4)			
Age, median (IQR)	61 (50–66)	62 (52–69)			0.849
**Indication for craniectomy**					
Stroke, *n* (%)	18 (41.9)	14 (50)	0.72	(0.27–1.87)	0.333
Traumatic brain injury, *n* (%)	11 (25.6)	6 (21.4)	1.26	(0.40–3.91)	0.458
Infection, *n* (%)	5 (11.6)	2 (7.1)	1.71	(0.30–9.49)	0.426
Hemorrhage, *n* (%)	9 (20.9)	5 (17.9)	1.21	(0.36–4.10)	0.501
Other, *n* (%)	0	1 (3.6)			0.394
**Sex**					
Female, *n* (%)	18 (41.8)	8 (28.6)	1.8	(0.65–4.98)	0.188
BMI, median (IQR)	25.4 (22.2–29.4)	26.8 (24.3–30.0)			0.494
ASA, median (IQR)	3 (3–3)	3 (3–3)			0.999
**Baseline mRS**					
1–2, *n* (%)	14 (32.6)	9 (32.1)	0.98	(0.35–2.71)	0.590
3–4, *n* (%)	21 (48.8)	15 (53.6)	1.10	(0.42–2.85)	0.518
5, *n* (%)	8 (18.6)	4 (14.3)	0.72	(0.19–2.6)	0.446
Time between first and second surgery, median (IQR)	115.5 (96.8–188.5)	116 (99–172.5)			0.745
**TMT prior to craniectomy, median (IQR)**	5.3 (4.4–5.8)	4.6 (4.2–5.4)			0.03
>5 mm, *n* (%)	29 (67.4)	9 (32.1)	4.37	(1.58–12.1)	0.003
≤5 mm, *n* (%)	14 (32.6)	19 (67.9)			
**TMT prior to cranioplasty, median (IQR)**	4.6 (3.9–5.5)	4.2 (3.6–4.7)			0.886
>5 mm, *n* (%)	19 (44.2)	5 (17.9)	3.64	(1.16–11.37)	0.019
≤5 mm, *n* (%)	24 (55.8)	23 (82.1)			
Muscle thickness reduction between craniectomy and cranioplasty, *n* (%)	32 (74.4)	25 (89.3)	0.34	(0.08–1.34)	0.106
**CSF shunt surgery, *n* (%)**	12 (27.9)	7 (25)	0.86	(0.29–2.54)	0.505
Simultaneous, *n* (%)	1 (2.3)	2 (7.1)	3.23	(0.27–37.43)	0.341
Pre-existing sinking flap syndrome, *n* (%)	4 (9.3)	0			0.127
Operative duration (min), median (IQR)	104.5 (87.3–135)	109 (90–147.5)			0.995
Decompressive craniectomy area (cm^2^), median (IQR)	95.8 (84.9–103.6)	97.6 (78.3–111.9)			0.661
**Implant material**					
Autologous bone graft, *n* (%)	34 (79)	25 (89.3)	2.20	(0.54–8.99)	0.214
PEEK implant, *n* (%)	5 (11.6)	3 (10.7)	1.09	(0.24–5.01)	0.611
Titanium implant, *n* (%)	1 (2.3)	0			0.605
Synthetic implant (other alloplastic material), *n* (%)	3 (7.1)	0			0.216
Acetylsalicylic acid therapy, *n* (%)	18 (41.8)	11 (39.2)	0.89	(0.34–2.37)	0.513
Continued therapy prior to cranioplasty, *n* (%)	8 (18.6)	10 (35.7)	0.41	(0.13–1.22)	0.091
Antiepileptic drug therapy	30 (69.8)	24 (85.7)	0.38	(0.11–1.33)	0.103
Tracheostomy prior to cranioplasty	22 (51.1)	13 (46.4)	0.82	(0.31–2.14)	0.441
Alcohol abuse	5 (11.6)	5 (17.9)	0.61	(0.15–2.31)	0.343
Nicotine abuse	19 (44.2)	9 (32.1)	1.67	(0.61–4.52)	0.222
Type 2 diabetes mellitus	7 (16.2)	5 (17.9)	0.89	(0.25–3.15)	0.553
Obesity	8 (18.6)	6 (21.4)	0.83	(0.25–2.74)	0.499
Urinary tract infection, *n* (%)	6 (13.9)	2 (7.1)	0.47	(0.08–2.53)	0.315
**mRS at discharge**					
1–2, *n* (%)	11 (25.5)	9 (32.1)	1.37	(0.48–3.93)	0.596
3–4, *n* (%)	23 (53.4)	13 (46.4)	0.75	(0.29–1.95)	0.367
5, *n* (%)	9 (20.9)	4 (14.2)	0.62	(0.17–2.28)	0.352
6, *n* (%)	0	2 (7.1)			0.152

Abbreviations: OR: odds ratio; IQR: Interquartile range; TMT—Temporalis muscle thickness; BMI—Body Mass Index; mRS—modified Rankin Scale.

**Table 3:** Multivariable logistic regression analysis of predictors for complication-related reoperation.

	Multivariate		
	OR	(95% CI)	*p*-Value
Age	1.02	(0.97–1.06)	0.478
Female	0.38	(0.12–1.15)	0.086
Baseline mRS	1.12	(0.76–1.66)	0.570
Continued ASA therapy prior to cranioplasty, *n* (%)	1.40	(0.49–3.98)	0.532
TMT prior to craniectomy (per mm decrease)	2.08	(1.16–3.7)	0.014

Abbreviations: OR: odds ratio; TMT—Temporalis muscle thickness; mRS—modified Rankin Scale.

**Table 4:** Type of postoperative complications requiring surgical revision after cranioplasty. Percentages in the “Count” column are calculated using the entire complication cohort, and percentages in the “Early complication” (<30 days; *n* = 11) and “Late complication” (>30 days; *n* = 17) columns are calculated using the corresponding subgroup denominators.

	Count	Early Complication	Late Complication
	*n* = 28	11 (39.3)	17 (60.7)
Bleeding or hematoma, *n* (%)	7 (25)	7 (63.6)	0
Postoperative cerebral edema, *n* (%)	2 (7.1)	2 (18.2)	0
Dislocation of the flap, *n* (%)	1 (3.6)	1 (9.1)	0
Infection, *n* (%)	8 (28.6)	1 (9.1)	7 (41.2)
Aseptic bone necrosis, *n* (%)	8 (28.6)	0	8 (47.1)
Wound healing disorder, *n* (%)	2 (7.1)	0	2 (11.7)
**Surgical intervention**			
Re-craniectomy, *n* (%)	14 (50)	4 (36.4)	10 (58.8)
Revision, *n* (%)	8 (28.6)	7 (63.6)	1 (5.9)
Conversion to alloplastic implant	6 (21.4)	0	6 (35.3)

## Data Availability

The datasets generated during and/or analyzed during the current study are available from the corresponding author on reasonable request as well as subject to institutional and ethical regulation.
